# Animal Models and Animal Experimentation in the Development of Deep Brain Stimulation: From a Specific Controversy to a Multidimensional Debate

**DOI:** 10.3389/fnana.2019.00051

**Published:** 2019-05-28

**Authors:** Sonia Desmoulin-Canselier, Baptiste Moutaud

**Affiliations:** ^1^Centre National de la Recherche Scientifique (CNRS), Nantes, France; ^2^Droit et Changement Social, UMR 6297, Université de Nantes, Nantes, France; ^3^Laboratoire d’ethnologie et de sociologie comparative, UMR 7186, Université Paris Nanterre, Nanterre, France

**Keywords:** animal experimentation, controversy, deep brain stimulation, history of neuroscience, innovation, translational medicine, Parkinson’s disease, psychiatry

## Abstract

In this article, we explore a specific controversy about animal experimentation and animal models in the recent history of deep brain stimulation (DBS), and we question its ramifications. DBS development intertwines clinical practice with fundamental research and stands at the crossroads of multiple legacies. We take up the various issues and controversies embedded in this rarely addressed dispute, from a standpoint that combines socio-anthropological and legal aspects. Our starting point is a debate on the role of animal experimentation in the development of DBS between Jarrod Bailey, a researcher promoting the abolition of animal experimentation, and Alim Louis Benabid, Marwan Hariz, and Mahlon DeLong, three key figures in the area of DBS and neuroscience. By clarifying the positions of the different protagonists and retracing the issues raised in these discussions, our objective is to show how this specific debate has extended from its initial space and how it provides an object of study with heuristic scope. We first present this partially polemic discussion about the history of DBS, and its link with a more general debate on the validity and use of animal models and the need for animal experiments. Then, we raise the issue of the relations and interactions between experiments on animals and on humans in the logics of biomedical innovation. The third step is to situate the discussion within the wider framework of opposition towards animal experimentation and the promotion of animal’ rights. Finally, combining these interweaved issues, possible implications emerge regarding the future of DBS. We show that behind these several controversies lie the question of translational research and the model of medicine upheld by DBS. We describe how the technology contributes to blurring the lines between research (fundamental, preclinical and clinical research) and care, as well as between humans and animals as substrates and objects of knowledge. The dynamics of DBS future development might then become a point of convergence for neuroscientists and animal rights defenders’ interests.

## Introduction

Deep brain stimulation (DBS) appears to be one of the major neuroscientific therapeutic innovations of the last 30 years (Sackeim and George, 2008; Insel, 2013)^1^. Considering its therapeutic effects and the scientific perspectives it opens up, the technology is stirring the interest of public policies (Gorman, 2013), industry (Aranzazu and Cassier, 2019) and the media (Racine et al., 2007; Gilbert and Ovadia, 2011)^2^. High-frequency DBS delivers electrical impulses to specific areas of the brain by way of implanted electrodes connected to a battery-operated neurostimulator unit placed in the chest. Producing spectacular effects on the motor symptoms in essential tremor and Parkinson’s disease, DBS offers treatment perspectives for serious, treatment-resistant disorders in neurology and psychiatry (Lozano et al., 2019). Its history intertwines clinical practice with fundamental research and industrial development and stands at the crossroads of multiple legacies that are difficult to dissociate in terms of practices and techniques (Gildenberg, 2005; Coffey, 2009; Hariz et al., 2010; Gardner, 2013; Moutaud, 2016; Aranzazu and Cassier, 2019; Dupont, 2019). The aim of this contribution is to question the place of animal experimentation and animal models in the light of a specific controversy and its ramifications, from a perspective combining sociology, social anthropology and legal issues.

This approach appears to be instructive for two reasons. First, it is interesting to explore the history of DBS from this angle, as it is told by its protagonists—the story of a therapeutic success which is also a successful example of interaction between therapeutic experimentation and clinical, preclinical and fundamental research. The role of animal experimentation in the development of DBS can also be apprehended from the angle of the sociology of techno-scientific controversies. It provides an object of study with heuristic scope to shed light on the complexity of controversies. Our case study is a debate that cannot indeed be rendered in an unequivocal and one-dimensional manner. Behind the controversy on the centrality of the part played by animal experimentation in DBS history lie many unfolding controversies involving protagonists with a variety of profiles, expectations and approaches.

The starting point to this reflection is a series of sharp exchanges in letters to editors between Jarrod Bailey, and Alim Louis Benabid, Mahlon DeLong and Marwan Hariz, that took place in 2014 and 2015 in the columns of the journal* Alternatives to Laboratory Animals* (ATLA) (Bailey, 2014, 2015a,b; Benabid et al., 2015a,b). Jarrod Bailey is a researcher involved in different activist organizations and associations promoting the abolition of animal experimentation. Alim Louis Benabid and Marwan Hariz, neurosurgeons, Mahlon DeLong, a neurologist and research scientist, are three key figures in the area of DBS and contemporary neuroscience. Their discussion is mainly focused on the place of animal models in the development of DBS. We will present this below in more detail. The debate drew our attention because it was an unusual dialogue between protagonists who seldom engage in public debates with each other: physicians and neuroscientists, on the one hand, detractors of animal experiments and defenders of animal rights on the other, even if certain neuroscientists also actively champion animal rights. These articles and letters to editors are also situated at the convergence of two different types of controversy: a contradictory and partially polemic discussion about the history of DBS, and a more general debate on the validity and use of animal models and on the need for animal experiments. This debate does not merely constitute the backdrop to a specific discussion. As we will show, the focus of the debate has spread out from its initial space and has found resonance beyond.

The characterization of these exchanges can, of course, be questioned. Is there really a controversy? Can there be controversy when the protagonists belong to such distinct spheres? This questioning is akin to an issue that has structured an entire field of the sociology of science, with the idea that a controversy can involve a variety of actors and arguments from outside of the scientific field. These actors can then fuel the debate with economic, environmental, political, legal, regulatory or ethical issues. In this sense, a controversy is a tool for a different approach to the history of science and the role played by contradiction in scientific development (Cambrosio and Limoges, 1991; Lemieux, 2007; Callon et al., 2009). Discussions around the role and the relevance of animal experimentation in DBS and of the conception of animal models for the pathologies concerned appear as an example of an “impure” controversy. This impurity—in reference to the heterogeneity of both the protagonists and the argumentative registers—opens the way to a wealth of interpretations by showing the content of the oppositions at work in the different social groups. From this angle, the discussion between Bailey and Benabid/DeLong/Hariz appears as a heuristically charged episode. This episode is indeed emblematic of what is at stake for the promoters of DBS (and promoters of neuroscience more broadly) in the production of a narrative and argumentation about their practices when these are called into question. This episode also provides a wealth of information on the constitution of arguments and the circulation of protagonists in the world of animal advocates and opponents of animal experimentation. Given this perspective, the issue is not so much to reproduce the sequence of events as to grasp the implications of the controversy for the modes of production of knowledge and innovation in DBS. The objective of this interdisciplinary contribution involving legal issues, sociology and social anthropology is thus to shed light on the different dimensions of the controversy, by presenting the positions of the different protagonists and the issues raised in the discussions, and at the same time to place what may seem a micro-debate in a wider context. The purpose of this article is then to study this unusual dialogue and to pull the threads of this debate to find out how it occurs between various social circles and within a complex environment.

To do so, a systematic literature review on animal model and animal experimentation in neuroscience would have been both useless and not sufficient. We focused instead on the issues raised by the debate between Bailey and Benabid/DeLong/Hariz and we systematically tracked down their sources in the literature, or in the literature related to the protagonists’ trajectories and their scientific and intellectual network. Our study was then based on three different sources: starting from the publication and *letters in response* between Bailey and Benabid/DeLong/Hariz, we first extended the analysis to a review of the scientific literature on the history of DBS and the place of animal models and animal experimentation in the first human DBS experiments, in neurology and psychiatry (for Parkinson disease, obsessive compulsive disorder, depression, and addiction)^3^. We then explored sociological and legal literatures, and legal sources (legal texts and court decisions) linked to animal models and animal experimentation in biomedicine and neuroscience, as well as sources for animal rights associations^4^. To complete these data, 12 semi-structured interviews were conducted with researchers and clinicians involved in DBS or/and animal experimentation in Europe (France, Germany, Belgium, England) and North America (USA, Canada) between 2014 and 2016. The purpose of these interviews was to understand more precisely and directly from the actors the internal reasoning and logics of their practices^5^. These interviews were only used as complementary sources of information (no software analysis was needed). The present work was also made possible by previous ethnographic research carried out by B. Moutaud in a neuroscience team developing DBS and animal experiments (Moutaud, 2015, 2016).

We will start by describing the exchanges between Bailey and Benabid, DeLong, and Hariz in order to present the debate on the role of animal models in the history of DBS (1). We will then raise the issue of the relations and interactions between experiments on animals and experiments on humans in the logics of biomedical innovation (2). The third step will place the discussion in the wider framework of the opposition towards the experimental use of animals and the promotion of animal rights (3). Finally, combining these three issues, possible implications emerge regarding the future of DBS and the dynamics of its future development (4).

## What Role for Animal Experimentation in The History of DBS for Parkinson’s Disease?

### The Discussion Between Bailey and Benabid, DeLong, and Hariz

Understanding the precise role of animal experiments in the history of DBS (to trigger or confirm therapeutic indications) requires closer examination of the research. Animal experiments can be involved at four stages in the development of DBS: fundamental research on animals concerning the anatomy and physiology of the brain structures and/or the physiopathology of the disorders in which DBS is to be tested; preclinical animal experiments to test DBS on an animal model of the disorder in order to assess DBS safety and therapeutic effect; DBS experimentation on animal models to understand the mechanisms underlying its therapeutic effects; the assessment of new devices or the optimization of stimulation parameters in an indication already experimented on humans. The role of animal experimentation in the development of DBS for Parkinson’s disease, in particular in the choice of the cerebral targets to be stimulated, did not appear as open to debate—or at least was not discussed until 2014.

Portraying the development of DBS for Parkinson’s disease and essential tremor as a success story has often involved a simplistic and imprecise chronology (see Gardner, 2013; Moutaud, 2016)^6^. This chronology started in 1986 when Alim-Louis Benabid discovered that high-frequency stimulation (over 80–100 Hz) of the thalamus had the same effect as the surgical lesion that was then used as a treatment for tremors: it stopped the tremors, which was at that time counter-intuitive. After having repeated the experiment on a number of patients, Benabid and his colleagues wondered whether high-frequency stimulation could be a good alternative to the stereotactic lesioning approach (Benabid et al., 1987). With his team in Grenoble, he implanted his first patients a few months later (Benabid et al., 1987). Experimental studies on animals enabled the validation of the subthalamic nucleus (STN) target. In 1990, Bergman and DeLong’s John Hopkins hospital team, using an animal model of Parkinson’s disease—MPTP monkeys—experimentally demonstrated the role of the STN (its hyperactivity) in the pathophysiology of the disease (Bergman et al., 1990). MPTP is a neurotoxin (1-methyl-4-phenyl-1,2,3,3,6-tetrahydropyridine) that causes symptoms similar to those of Parkinson’s disease by destroying the substantia nigra dopaminergic neurons (in humans, Parkinson’s disease motor symptoms result from the progressive death of dopaminergic cells in the substantia nigra). This animal model contributed to establish that a lesion of the STN led to an improvement in Parkinson’s symptoms induced in these monkeys. Following these results, in 1993 a team from Bordeaux (Benazzouz et al., 1993) showed the efficacy of high-frequency electric stimulation in the STN in two MPTP monkeys. This was a turning point in the application of DBS to Parkinson’s disease. The STN, described previously as a “no man’s land” by neurosurgeons, and a delicate target to reach, with a risk of serious adverse events (abnormal movements, such as hemiballismus or dyskinesia; Wichmann et al., 2018), became from then on the principal target in DBS indications for Parkinson’s disease.

This success story links Benabid’s exploratory discoveries, the development of the MPTP monkey model and the experimental demonstration of STN stimulation effectiveness on these animals. The story continues with the organization of clinical trials on large cohorts, the marketing authorization for the medical device (European Union certification and approval from the American Food and Drug Administration), followed by registration on the list of acts and products eligible for reimbursement in different countries.

However, Bailey disagreed with this narrative. In an article dedicated to the implications of genetic differences between monkeys and humans in animal experimentation published in ATLA in 2014, he briefly evoked DBS. He wrote: “Deep-brain stimulation (DBS) often claimed to have been developed through critical NHP (non-human primates) experiments, was actually discovered serendipitously in a human patient and owes nothing to NHPs for its advancement” (Bailey, 2014: 246). His argument, developed in later publications, highlights the fact that experimenting on monkeys was not compulsory (Bailey, 2015a,b; Bailey and Taylor, 2016). According to him, most phenomena demonstrated in monkeys had already been described in humans long before, as early as the 1950s. Following his reasoning, a deeper knowledge of the data and a more refined exploration of these leads, directly applied to humans with the help of contemporary technologies (brain imaging in particular) would have led to the same breakthroughs. He explained that several teams had already reported the inhibition of tremors by electric stimulation in the thalamus or the STN in similar operative conditions. In addition, he claimed that, for Parkinson’s disease, DBS in the STN had already been experimented before Benabid. In his 2015 letters to the editor, Bailey criticized Benabid, DeLong and Hariz for overlooking a whole section of the history of research and discoveries dating long before Benabid, which would have enabled monkeys to be spared (Bailey, 2015a,b).

In their letters in response, Benabid, DeLong and Hariz decry the terms of the debate as distorted (Benabid et al., 2015a,b). For them, Bailey used the scientific literature in a flawed way. They claimed he confused not only electric stimulation and lesion/ablation, DBS of the ventral intermediate nucleus (VIM) and DBS of the STN, but also distinct cerebral regions (STN and structures situated around the STN). Furthermore, he seemed to forget that the link with the high-frequency parameter had never been made. And according to them, it was the evidencing of the inhibiting effect of high-frequency stimulation that constituted the crucial nature of Benabid’s discovery of 1986/1987. This discovery opened a new approach through research on animals, where the STN was the preferred target. Before that, no neurosurgeon would have dared to intentionally cause a lesion or implant electrodes in the STN in the current state of knowledge.

### Several Circles of Controversies

The comparison and confrontation of the arguments of Bailey and Benabid/DeLong/Hariz enable us to apprehend the mutual incomprehension. The neuroscientists stress the need for a precise location and technical approach, pointing out that a real discovery occurred in the late 1980s on the strength of animal experiments, whereas the animal rights advocate opts for a broader picture, tempering the importance of the contribution by Benabid and DeLong, postulating that animal experiments were not necessary in view of the data already available (from cumulated data on stereotactic lesioning and electrical approaches on various brain targets). Another element of comprehension could also be, in the background, a blur effect created by the simplistic narration found in numerous scientific articles on DBS, thus giving a deceptive impression on two fronts: Benabid’s discovery was presented as being felt like a clap of thunder in the calmness of the sky, and animal experiments as having been developed from the very first phase of the DBS development process. There is however an unanswered question. Indeed, the quotation cited above was only two lines from a 30-page article on genetic research written by Bailey, who signed as a member of an association protesting against animal experiments (the *New England Anti-Vivisection Society*). Why did three prestigious neuroscientists feel the need to provide an answer to these two lines in a journal devoted to alternative methods? In our view, three potential explanations or levels of interpretation can be suggested.

First, their responses to Bailey could be understood as merely a means to re-establish scientific and historical facts.

Second, an explanation could be found in what was at stake in this narrative. In fact, these exchanges occurred in 2014, a few months after Benabid and DeLong were awarded the *Lasker-DeBakey Clinical Medical Research* prize for their respective work and contributions to the development of DBS for Parkinson’s disease^7^. Bailey’s article, therefore, appeared at a key moment and challenged a narrative that had been accepted and legitimized by one of the most prestigious awards in fundamental and clinical research. Alongside, the same preoccupation about the recognition of the “essential” or “crucial” role of animal experimentation and their own studies in the history of DBS was formulated by Abdelhamid Benazzouz and his team in several publications that came out after the Lasker prize had been announced (Benazzouz et al., 2016; Benazzouz, 2017; Faggiani and Benazzouz, 2017).

Third, it is probable that neuroscientists’ perceptions of the rising protest against animal experiments had a role in this “controversy.” Indeed, for Bailey (and other authors presented below) the debates extended more widely and the stance had a clearly normative aim. By “dismantling” the discourse on the usefulness of animal experimentation, its opponents are trying to denounce the relevance of animal experimentation and animal models in biomedicine, in order to eventually obtaining their prohibition. It is hard to imagine that (at least) DeLong who has been conducting animal experiments since the 1970s, was unaware of the debate surrounding this practice and the rising power of the anti-vivisectionist movement. The fact that he co-published letters in response in a journal devoted to experimental alternatives at the time when a European citizen initiative (discussed hereafter) was submitted to the European Commission to prohibit animal experiments, reinforces this interpretation. The publication of a letter to the editor in this journal at this particular time leads us to believe that the “controversy”—if indeed it is accepted as such—was developing beyond ATLA^8^.

These interpretations are not necessarily alternative but rather complementary. They suggest that the exchanges between Bailey and Benabid, DeLong and Hariz are embedded in other controversies. Behind the debate on how the history of DBS should be told and written (with an often central question as to what was discovered or invented by whom, when and how: Moutaud, 2016), another question arises concerning the logics of research and innovation and the legitimacy of animal experiments. Indeed, these discussions also raise the issue of DBS as both a therapeutic tool and a research technology. As Benabid, DeLong and Hariz pointed out on the development of DBS in the STN, with direct reference to the Lasker prize: “The huge impact of this research has resulted from a continuous interaction between human and animal research (Benabid et al., 2015a: 205).” Besides the opportunity to re-explore the history of DBS and to place Benabid’s work in a different historical depth, these readings of the role of the MPTP animal model and animal experiments stress the importance of to-and-fros between experiments on humans and animals, therefore pointing to DBS as a model of translational research.

## Why Is DBS A Good Illustration of The Translational Paradigm?

In western biomedicine, the classic logics of ethical and legal discourse since the 1970s support a linear model of development corresponding to a fundamental-preclinical-clinical research sequence. In this perspective, animal experiments are thus not only authorized but also seen as a prerequisite to experiments on humans^9^. These principles are present, more or less explicitly, in texts with variable normative value, such as those of the Belmont Report (Ethical Principles and Guidelines for the Protection of Human Subjects of Research, The National Commission for the Protection of Human Subjects of Biomedical and Behavioral Research, April 18, 1979), the World Medical Association’s Declaration of Helsinki (Ethical principles for medical research involving human subjects, 1964 modified), the Oviedo Convention on Human Rights and Biomedicine of April 4th 1997, or the European Union regulation n°536/204 of April 6th 2014 on clinical trials on medicinal products for human use.

In France, for example, animal experiments are in principle prerequisites to any biomedical experiments on humans, as confirmed by the *Code de la santé publique* (French Public Health Code). It is indeed with reference to experiments on animals that article L.1121-2 states that “no research involving a human being can be carried out if it is not founded upon the latest scientific knowledge and on adequate preclinical experiments^10^.” In addition, texts relating to the evaluation of research projects (conditioning administrative authorization), by the French *Comités de protection des personnes* (Committees for the Protection of Human Subjects), must include reference to the “prerequisites,” i.e., data collected in the chemical, toxicological, biological, pharmaceutical, clinical domains, which also entails the conduct of experiments on laboratory animals. Although, in contrast with what was initially planned by a bill that led to the Huriet-Sérusclat law of December 20th 1988, the requirement to resort to animal experiments before any experiment on humans is not actually specified, it nevertheless pervades ethical and legal conceptions^11^. It is, therefore, the fundamental-preclinical-clinical research sequence followed by treatment that constitutes the frame of reference. Treatment is thus merely perceived as an application of what has first been tested on animals and then on humans.

Yet DBS was not initially tested on animals in all indications. For certain applications, such as essential tremor and Parkinson’s disease (for the VIM target), Tourette’s syndrome or psychiatric disorders such as obsessive-compulsive disorder (OCD) and depression, experiments on humans preceded the resort to animal experimentation (Feenstra and Denys, 2012). Depending on target and pathology, animal models may or may not have been available, and in some cases, they were developed in parallel or following the first experiments on humans for the purpose of more in-depth and refined exploration of the therapeutic hypotheses and leads. This seems to challenge the ethical and legal frame of reference. But, on closer examination, international and national legal texts are not so explicit and unequivocal about the necessary prior use of animal experimentation, apart from regulatory procedures for drug marketing. This allows uncertainty to persist on the interpretations to retain outside this particular field. Implantable cerebral devices do not come within the legal scope of drugs and medication, and clinical trials do not account for the whole of biomedical research (Adèle and Desmoulin-Canselier, 2016; Desmoulin-Canselier, 2018). Furthermore, even though standard clinical trials serve as models, they do not encompass all the different forms of experimental studies. In DBS, there has been considerable resort to case studies and small-size cohort studies with a “proof-of-concept” objective (Schlaepfer and Fins, 2010; Moutaud, 2016). Moreover, the model of randomized controlled clinical trials have been contested, in particular, because it was designed for drug market authorization, whereas surgically implanted medical devices could require alternative criteria of proof (Fins et al., 2017; Moutaud and Aranzazu, 2019).

Our review of the literature and interviews with researchers confirmed that animal experiment protocols are often deployed in parallel and in a back and forth movement with research and sometimes with experimental therapeutic applications on humans. In this sense, DBS appears as an illustration of the translational research/medicine concept, which aims to reinstate circularity and porosity between these different stages and different registers (Bréchot, 2004; Desmoulin-Canselier, 2015; Cambrosio et al., 2018). To-and-fros between research and treatment, and between experiments on humans and experiments on animals, are liable to follow different paths. To demonstrate this, DBS applications to OCD and addictions can be mentioned here.

In the case of the first application of DBS to OCD by a Belgian team from Leuven University at the end of the 1990s (Nuttin et al., 1999), participants in the clinical trial were implanted in the same brain region than for stereotactic lesioning (the anterior limbs of the internal capsule). As a justification, the team put forward the same arguments as for DBS in Parkinson’s disease: compared to stereotactic lesioning, DBS seemed less invasive because it was “reversible” and “adaptable” and entailed potentially fewer adverse events (Nuttin et al., 2000; Gybels et al., 2002). Since then, the team has nevertheless stepped up their research on OCD animal models (rats) in order to fine-tune their choice of cerebral target and to address certain limitations of their initial research (van Kuyck et al., 2003). This instance, therefore, echoes that of DBS for essential tremor and Parkinson’s disease mentioned earlier: an application of DBS deployed for its advantages compared to stereotactic lesioning, with experiments on animal models occurring afterward to optimize practice.

In 2004, a clinical trial was developed in France for assessing DBS efficacy for the treatment of OCD (Mallet et al., 2008). The rationale of this research was also supported by to-and-fros between humans and animals but this time with a detour through a treatment situation. The original choice of the brain target (the STN) was justified by unexpected therapeutic effects observed in two parkinsonian implanted patients who had seen their comorbid OCD symptoms reduced (Mallet et al., 2002). At the same time, this research team developed an animal model of stereotyped and repetitive behaviors in monkeys in order to explore the effects of DBS (Grabli et al., 2004; Baup et al., 2008). This research was developed too late to be part of the scientific and ethical legitimization of the OCD clinical trial, it was nevertheless used later to explain the effects of DBS on OCD symptoms and to initiate new indications and new targets in humans (Haynes and Mallet, 2010; Moutaud, 2015).

DBS development for the treatment of addictions (alcohol, heroin and cocaine) conveys different but complementary information. These DBS indications also stemmed from observations in treatment contexts. Some patients treated with DBS for Parkinson’s disease, OCD or Tourette’s syndrome developed compulsive behaviors (pathological gambling, alcohol, nicotine or dopaminergic treatment addictions, etc.) considered to belong to the same spectrum of disorders as that of addictions, while others felt an improvement for similar disorders (Carter and Hall, 2011; Luigjes et al., 2012; Pelloux and Baunez, 2015). These observations suggested a possible effect of DBS on addictive disorders. The chosen targets are also often derived from experiments on humans in stereotactic lesioning. However, in this case, research teams also develop preclinical research on animals (mostly rodents). For trials in the field of addiction, in western countries, DBS has been used on humans only after a study on animals had been carried out (Pelloux and Baunez, 2015). Could the controversial context of this indication (a surgical treatment for addictions) explain a return to the linear model of preclinical to clinical research? It is interesting to note, however, that the ethical debate concerning the first psychiatric indication (OCD) started *after* the first experimentations on humans (Moutaud, 2014), whereas for addictions the ethical debates preceded applications on humans (Carter and Hall, 2011).

Despite the heterogeneity of the cases described, and even if the division between experimentation and therapy is often a fine line in medicine, one observation can be made: DBS blurs the paradigmatic lines established since the 1979 Belmont Report between fundamental, preclinical and clinical research and treatment (Largent et al., 2011). Since the 1980s, DBS indications have been developed not only from previous knowledge acquired in clinical practice and fundamental research, but also from the back and forth between DBS experiments on humans and animals, single case or small cohort studies, double-blind randomized clinical trials and serendipitous observations in therapeutic situations (Schlaepfer and Fins, 2010; Moutaud, 2011, 2016; Fins, 2012; Hariz, 2012; Gardner, 2013). It constitutes a form of translational treatment, and the role animal models have in its development highlights this situation particularly well. It may not be the only example of this kind of disturbance, but it is a recent (in the post Belmont Report era) and remarkably informative one. The Bailey vs. Benabid, DeLong, and Hariz “controversy” points to the nature of DBS as a technology of treatment and research and as a tool to produce evidence or “proof” of efficacy (Fins, 2012). The specific characteristics of DBS—its “reversible” and “adaptable” nature, and the conditions of its surgical implantation and the fine-tuning of stimulation parameters—enable a direct exploration of human brain functioning in treatment or research situations (Moutaud, 2016). It is also for this reason, and because the human brain remains the substrate of the individual, that DBS is an interesting example for the detractors of animal experiments. Following this thread, we will now trace the “controversy” in a new direction: the debate on the validity of animal models, and on the legitimacy of animal experimentation.

## Is The Issue of Animal Experiments in DBS A Controversy Specific to The Field?

### Contesting Animal Experiments

The discussion concerning the role of animal experimentation in the history of DBS is set in a wider controversy about the scientific validity and usefulness of animal experiments. This debate extends into two complementary directions. First, it leads to the contestation of animal models’ ability to mimic human pathologies and to verify the innocuousness and efficacy of health products. Second, it opens up contestation of the use of animals with regard to their cognitive abilities and phylogenetic proximity with humans, which have notably been demonstrated through neuroscience research.

To understand how these controversies and different arenas of discussion are articulated, we shall return to the exchanges published in ATLA. This scientific peer-reviewed journal aims to circulate research results on alternative methods to animal experiments. Bailey is a geneticist and he positions himself first of all in the field of the implications of inter-species and inter-individual genetic differences to contest the scientific relevance of using animal models to validate human therapies. This “insider” protest is however at odds with what could be a “scientist-to-scientist” debate. Indeed, Bailey signed the 2014 article as a member of the *New-England Anti-Vivisection Society*—which contests the relevance of animal experimentation—and he did not hesitate to deviate from his field of expertise to talk about DBS. Nevertheless, his criticism of animal experiments is mainly conceived and presented as scientifically sound.

Bailey, first of all, raised the issue of the validity of animal models and the scientific relevance of animal experiments. To support his viewpoint on the history of DBS, he referred to two articles signed by neuroscientists: Lawrence A. Hansen, a specialist in neuropathologies (Greek and Hansen, 2012), and Marius Maxwell, a neurosurgeon (Maxwell, 2009). However, in doing so, Bailey was not referring to an internal quarrel in the neuroscientific world. Instead, he linked his own reflections to the cross-sectional controversy on the scientific justification of animal experiments. The article co-signed by Hansen on the role of animal models in the development of DBS was published in collaboration with Ray Greek (Greek and Hansen, 2012). Greek is a physician specialized in anesthesiology who had previously co-authored with Niall Shanks a well-known article among anti-vivisectionists claiming the absence of a predictive value for results derived from animal models (Shanks et al., 2009). As for Shanks, he is a philosopher of science and one of his articles co-written with Hugh LaFollette is a reference among animal experimentation opponents, for shedding light on the philosophical terms of the debate concerning the usefulness and scientific relevance of resorting to animal models (LaFollette and Shanks, 1995). Concerning Maxwell (2009), Bailey’s second reference, his publication was an open letter published on the Website *Voice for Ethical research at Oxford* (VERO), an association opposing animal experiments. The arguments presented in support of the creation in 2006 of this association were that the United Kingdom *Animal Scientific Procedure Act* adopted in 1986 was supposed to lead to a progressive end of animal experiments, which is still not achieved today. Thus, Bailey’s references draw on two sources: criticism by neuroscientists, and a more general, pluridisciplinary criticism of animal experimentation. This shows that different registers interweave, and it is true that pluridisciplinary and pluri-motivated publications are frequent in the literature devoted to the contestation of animal experimentation.

The 2014 exchanges in ATLA are therefore part of a broader circle of discussion. They were taken up in particular by Anne Beuter, a French neuroscientist in Bordeaux (now Emeritus Professor). In 2017 she published an article in ATLA in which she presents neurocomputational models as an alternative to animal experimentation in the development of neuromodulation treatments. In this article, she explicitly refers to the case of animal experiments in DBS and to the discussions between Bailey and Benabid, DeLong, and Hariz, and she supports Bailey’s position (Beuter, 2017). Anne Beuter is also scientific advisor to the association *Antidote Europe*, created in 2004 by Claude Reiss (cellular and molecular biology researcher), Hélène Sarraseca (neuroscientist) and André Ménache (a vet who also published with Ray Greek). In December 2015, she published a note in the *Antidote Europe* periodical to dismantle the “neuroscientific myth” attributing the discovery of DBS for the treatment of Parkinson’s disease to animal experiments and the MPTP monkey model (Beuter, 2015)^12^. It should also be noted that *Antidote Europe* plays an important part in contesting animal experiments and fighting legally for its abolition. For instance, it played a key role in the European citizen initiative “Stop vivisection!” in 2015 and it seems important to say a few words about it.

“Stop vivisection!” is the third initiative filed since the creation of the European citizen initiative procedure by the Lisbon Treaty, and it has collected 1.17 million signatures from citizens from the 26 member states^13^. Its aim was to obtain from the European Commission a project for the repeal of the 2010/63/EU directive (22 September 2010) relating to the protection of animals used for scientific purposes, and for a new proposal aiming to abolish animal experimentation. This purpose could appear paradoxical to those who believed that the objective was to improve outcomes for laboratory animals. However, “Stop vivisection!” project was to directly challenge the scientific validity of the approach consisting in resorting to animal tests to establish the safety of a product intended for humans, or to extrapolate results from animal experiments to human medicine. The objective was to obtain another regulatory system and another research approach: making “compulsory the use—in biomedical and toxicological research—of data directly relevant for the human species^14^.” The Commission turned down the request in 2015 (Desmoulin-Canselier, 2017) but it earned considerable publicity. The link with the ATLA exchanges is, of course, indirect, but it seems perfectly clear. Bailey and his publications (including his 2014 ATLA article) are referenced in the arguments that support the “Stop vivisection!” European citizen’s initiative^15^. Interviews with Bailey dating from 2006 and 2008 have also been put on the *Antidote Europe* website^15^.

In their respective publications, Bailey, Hansen, Maxwell, and Beuter mainly discussed the role of animal experiments in the development of DBS for Parkinson’s disease. However, the discussion also questions the scientific validity of the MPTP model. It is emphasized that the monkey species that are mostly used are phylogenetically distant from humans (African vervet monkeys, rhesus macaques and cynomolgus monkeys) and that the pathology is artificially induced by injection of the MPTP neurotoxin. The model is also criticized because it does not provide a faithful image of evolutive diseases: it does not reproduce the temporality of the degenerative process, the progressive loss of neurons and consequently, the variety of clinical phases that patients go through. It is a “snapshot” model of the disease. Moreover, a substantial percentage of monkeys spontaneously recover their motor capacities, which requires repeated injections (Fox and Brotchie, 2010; Porras et al., 2012). These characteristics make the MPTP model particularly artificial and debatable in the eyes of the detractors of animal experimentation. They are mentioned for instance in the arguments questioning the lawfulness of experiments carried out on MPTP monkeys in a Paris-based research center filmed in a controversial video broadcast by the association *Animal Testing*. Shot in 2013 and broadcast at the beginning of 2017, this video was circulated to other associations involved in the defense of animal rights (for example the *Fondation droit animal, éthique et sciences*: Bachelard, 2017). Animal experimentation in DBS was therefore at the heart of the discussions in France in 2017, at the same time as discussions were taking place on the revision of the above-mentioned 2010/63/EU directive, in particular for its provision concerning primates.

The debate here, however, takes on a whole new dimension. It is not solely a question—as in the “Stop vivisection!” initiative—of contesting the scientific validity of animal models, it also raises the issues of the living conditions and the very use of laboratory animals. Even though the registers of the arguments are conceptually distinct, there are nevertheless points of convergence^16^. Here, another type of controversy emerge: that of animal rights and their legal status. It is for primates, and especially for the great apes, that demands for a change in legal status appear as the most advanced in the legal field. Concerning legal texts, the 2010/63/EU directive contained an unprecedented specific regime that was particularly protective for primates, on account of scientific knowledge on their cognitive abilities and their phylogenetic proximity with humans (Rémy, 2011). Animal rights advocates drew notably on scientific data from studies in the neurosciences and the cognitive sciences to emphasize the fact that humans and monkeys share similar “cerebral circuits” for language and communication and that they share the development of frontal lobes involved in choice and planning. Experimental results are thus re-appropriated and diverted from their initial objectives, whilst the analogical dimension of scientific studies on animals is highlighted. Florence Burgat, a French philosopher and major figure in the contemporary struggle for animal rights, stresses “the paradox that is internal to experiment” in the following way: “the essential psychophysiological proximity between men and animals is asserted, so that one species can stand for the other, but alongside, this proximity is denied in order to establish a relationship in which there is no reciprocity” (Burgat, 2009: 197)^17^. According to her, this paradox needs to be lifted.

The controversy on the legitimacy of animal experiments, therefore, entails a debate over the instrumentalization of beings for whom studies in neuroscience have shown their complexity and the existence of interiority. But it also involves the contestation of the animal model because of its artificial nature and its inability to produce transposable predictions due to inter-individual singularities (not only between humans and animals, but also between laboratory and non-laboratory animals) and difficulties in transposing from controlled laboratory conditions to patients’ real lives, or to other animals (Canguilhem, 1965; Gerber, 2016). This last point finds a particular echo in the field of psychiatry, where the specificity of human pathologies and the effects of transposition appear bluntly. Within this specific framework, the controversy takes a particular turn, as it can be clearly seen in the world of medical research. This supplementary stratum of “purely” medical and scientific controversy, presented below, is well known by animal rights defenders who use it in their own arguments.

### The Controversy Over Animal Models of Psychiatric Disorders

Animal models of psychiatric disorders do not escape criticism relating to the artificial dimension of induced disorders or symptoms that do not pre-exist in animals, even if this issue remains open for certain disorders such as depression (Krishnan and Nestler, 2011). However, the debate here takes root in a more fundamental doubt regarding the very possibility of conceiving animal models for human behavioral or emotional disorders (Nestler and Hyman, 2010). An enterprise of this sort raises acute epistemological issues, even in the neuroscientific community (Rose and Abi-Rached, 2013; Lemoine, 2015). Here, the debate on animal models is nurtured by discussions concerning the nature of psychiatric disorders, their definition, and the criteria and tools used to describe them. There is thus the question of the actual possibility of reproducing in an animal a pathophysiological phenomenon for which the causal process has still not been mapped out in humans, limiting from the outset the conceptual validity of the models (Nestler and Hyman, 2010). While animal manipulations are supposed to help identify these causes by reproducing the symptoms of the disorder, the model never expresses the whole set of symptoms. This limitation is then compounded by the issue of identifying with certainty various cognitive and emotional manifestations in animals, or the “typically human” subjective components that characterize these disorders (Feenstra and Denys, 2012: 218). Any choices in this field open up possible discussion on the continuity and the transposable nature of behaviors or cognitive and emotional states from animals to humans. These aporias lead researchers to concentrate these models on potentially objectifiable components, on prominent clinical traits of the disorders modeled, breaking them down and putting aside their psychopathological complexity (Moutaud, 2015). Rather than an animal model of a disorder, what is generally proposed is an animal model of a transnosographic symptom or behavior (attention, anxiety, stereotyped behaviors, etc.). The model thus developed can be cross-sectional to other pathologies sharing its prominent characteristics. It is nevertheless important to know what components of a psychiatric disorder should be modeled in order to “represent” it.

As evidenced in the literature (and explained by researchers during interviews), with DBS, anatomical, ethical, economic and technical constraints are added to these epistemological limitations. One can imagine that their accumulation has been the cause of certain particularities in the development of animal models for psychiatric applications of DBS. The application of DBS to treatment-resistant depression is a particularly telling case. It also sheds further light on the innovation processes and the place given to animal experiments in the development of DBS. Research on animals to exploring the effects of DBS on depression has mainly been carried out on rodents (Scott et al., 2012). It has either been conducted prior to clinical experiments on humans (to assess the anti-depressant effect of DBS), or in a circulation of the translational type with to-and-fros between humans and animals, in order for instance to refine cerebral targets or determine optimal stimulation parameters (Scott et al., 2012; Dandekar et al., 2018). These models are debated in the light of the epistemological limitations previously mentioned, but their transposability comes up against another limitation: the absence of correspondence between human and animal brain anatomies, and consequently for cerebral stimulation targets (Widge and Dougherty, 2015). This difficulty mainly concerns rodent anatomy, which makes the resort to monkey models more attractive. In addition, behavioral complexity is another major argument in favor of non-human primates for modeling psychiatric disorders. However, the possibility of focusing on primates entails ethical and legal limitations. Non-human primates are subject to more stringent regulations, and the 3R’s principle (for “replacement,” “reduction,” and “refinement”) is an ethical and legal requirement (at least in Europe) that constrains researchers’ choice regarding the kind of species used for experimentations. These ethical and legal limitations (including the need to take potential animal pain, suffering or distress into consideration) are also coupled with technical and financial obstacles (the animals and the adapted stimulation devices are costlier). Finally, with all animal species considered, animal experiments in DBS imply the development of specific devices. Indeed, the implanted device used for human patients cannot be directly used for animals and it has to be adapted to their morphology (Feenstra and Denys, 2012). This constraint produces a new potential source of bias for the transposition of results from animals to humans.

Is it for these reasons that most research on the antidepressant effects of DBS have been first conducted directly on humans (Scott et al., 2012; Dandekar et al., 2018)? Going back to the neurologist Helen Mayberg and the neurosurgeon Andres Lozano’s seminal research (Mayberg et al., 2005) on the application of DBS to resistant forms of depression, we observed that no preclinical animal experiments were mobilized to support its use, except for the functional exploration of cerebral structures targeted, or research on the physiopathology of the disorder. After the discontinuation of the first two double-blind randomized clinical trials in 2014 and 2015 funded by the industry to assess DBS efficacy for the treatment of depression, substantial debates took place to identify the causes of these failures: choice of cerebral targets, stimulation parameters, definition of the population, etc. (Schlaepfer, 2015; Mayberg et al., 2016; Widge et al., 2016, 2018). However, until recently, research on animal models was not mentioned among the potential leads to be followed (for instance: Bari et al., 2018). Interestingly, technological and methodological innovations and the possibility of exploring the phenomena in their complexity directly in implanted patients have aroused increased interest (Fins et al., 2017; Moutaud and Aranzazu, 2019).

Can this shift be interpreted as the progressive ending of animal experiments in the field of DBS in favor of research developed directly on humans and based on human data? In all events, the contestation of animal models and animal experiments finds promising perspectives in the new neuromodulation technologies.

## What Consequences for The Future of DBS Technological Innovation?

Beyond the discussion between Bailey and Benabid, DeLong and Hariz, different levels and circles of controversy have emerged. They concern both the history of DBS and the role of animal models in determining targets for diseases, the validity of animal models in neuroscience and biomedicine, and the legitimacy of the instrumentalization of animals. Alongside, it is the role of research directly carried out on humans that is highlighted. Could a point of convergence between the worlds of neuroscientists and that of animal rights defenders be found in the recent innovative approaches applied in DBS? This counter-intuitive convergence could stem from the coexistence of a contestation of animal models in biomedicine, technical progress in investigation technologies, and a movement towards an individualization of medicine, thus leading to a new inclination in favor of research directly carried out on humans.

As Enna and Williams pointed out, “a major hurdle in the translational medicine undertaking is the fact that most preclinical animal models of disease generally lack predictive value with respect to the human condition under study” (Enna and Williams, 2009: 12). Certain on-going developments in the field of DBS are based on the idea that the human brain appears so specific and presents such inter-individual variability that any progress needs to come from the direct exploitation of individual patient data. In this perspective, researchers/clinicians should resort to devices and technological systems enabling them to optimize data collection during surgical procedures and during clinical follow-up. They could derive information explaining the efficacy of DBS and use that information to adapt treatments.

A first example is provided by a clinical trial carried out by Helen Mayberg on ten people suffering from resistant depression, aiming to test a new stimulation electrode^18^. This electrode—which has the potential not only to stimulate but also to record brain activity in real time, both during the surgery and outside the operating room—is generating considerable expectations (Fischer, 2015). The potential knowledge gains concern both the pathophysiology of depression and the action mechanisms of DBS. This research follows on from previous work by Mayberg in neurophysiology and brain imaging (Mayberg et al., 1999; Mayberg, 2003; Choi et al., 2015) using data collected directly on humans. She justified the choice of the brain target (the Broadman Area 25) for the first DBS clinical trial for depression, and she distinguished categories of people suffering from depression according to their brain structural or functional profiles. Her research, therefore, offers an example of what can be seen as an innovating approach avoiding animal experimentation by exploring new ways of acquiring knowledge in neurology and psychiatry through new neurotechnologies (Moutaud and Aranzazu, 2019).

Another example is provided by the “closed-loop” technology. These DBS devices are being developed to continuously collect brain activity signals in the implanted patient and to adapt in real-time the stimulation to the patient’s clinical state (while devices used up to now only enable continuous stimulation according to pre-set parameters). This could enable a better understanding of the effects of DBS and the pathophysiology of the disorders. It also offers perspectives for personalized medicine with on-demand stimulation adapted to individual clinical and biological profiles. Anne Beuter, already mentioned, is among the promoters of this innovation. With her team in Bordeaux, Beuter has developed research on closed-loop technology for neuropathologies, and she supports the idea that this promising lead is the real future of neuromodulation (Beuter et al., 2014; Beuter, 2015; Ménache and Beuter, 2016). This neuroscientist clearly makes a connection between this opening for technological development and the contestation of animal experiments. According to her, these innovations could, on the one hand, enable animal models to be rendered dispensable by collecting and processing human data, and, on the other hand, accelerate the translational research process by articulating experiment with treatment in a single process.

This point of view and this tendency, however, need to be counter-balanced by other factors. First, studies on animals have also been developed to investigate and assess new devices, whether recording devices or closed-loop technologies (Johnson et al., 2016). Second, research on animals has been deployed in new directions, as is the case with optogenetics. Combining optics and genetics, this approach aims to pilot the activity in nerve cells that are genetically modified with a light beam. Presently carried out on rodents, this type of experiment seems to be a promising trend to determine cerebral targets for DBS or identify the pathophysiological mechanisms of diseases (Creed et al., 2015). Nevertheless, optogenetics is open to certain previously mentioned criticisms, such as the artificial nature of the experimental setup (genetically modified animals) (Akhtar, 2015). In addition, even if its purpose is therapeutic, optogenetics cannot be experimented with, at this time, on humans and can only be deployed for fundamental research (Williams and Denison, 2013).

Finally, these lines of innovation and research strategies in humans might raise several epistemological limits and legal and ethical concerns. Alternative methods such as closed-loop technologies are already subject to criticism concerning patients’ autonomy and their role in the decision-making process (Gilbert et al., 2018). Intensifying research on humans is probably not a generalizable outlook and these innovations might renew the debate over the legal and ethical regulation of neuromodulation devices, their safety, the validity of the data produced, and the porosity between experimentation and therapy in DBS (Bell et al., 2009; Schlaepfer and Fins, 2010; Fins et al., 2011; Moutaud, 2011, 2016; Blank, 2013; Desmoulin-Canselier, 2018). Nevertheless, as the topic arose in the controversy surrounding animal models and animal experimentation, within the history of DBS, it appeared important to describe how and why the actors situate these alternatives within the debate.

## Conclusion

This article has focused on the discussions that surrounded the mobilization of animal experiments and animal models in the recent development of DBS. We have shown that this particular debate opens up other controversies. Each of them has its own logic, but they converge in many ways. We have seen that behind the contestation of animal experiments and of the validity of animal models in the field of neuroscience lies the question of the research and treatment model upheld by DBS. The technology today meets the ambitions set out by public authorities and other protagonists in terms of “translational” medicine or research. As a result of its characteristics and its technical potential, DBS contributes to a blurring of the frontiers between fundamental, preclinical and clinical research and treatment in humans, between neurology and psychiatry, and also between humans and animals as substrates and objects of knowledge. It also gives substance to the broader neuroscientific intellectual and epistemological project to extend beyond the established disciplines (neurology, psychiatry, cognitive sciences, etc.).

Since the 1970s, teams involved in DBS development have been confronted with difficulties in producing, for a surgical technology, proof considered to be sufficiently robust, while the relevant assessment tools were designed for pharmacological research (Coffey, 2015; Fins et al., 2017; Moutaud and Aranzazu, 2019). These difficulties have recently led clinicians and researchers in DBS to question methods and tools of evidence-based medicine, thus motivating calls for methodological and technological innovation. In this context, they expect to draw on the advantages of DBS potentialities and to find new lines of innovation. This could then generate innovative practices, provide access to robust data and evidence, it could form an experimental framework, and foster serendipity (Fins et al., 2017). However, DBS could then open new debates on the scientific, legal and ethical framework and the regulation of neuroscience practices.

## Author Contributions

BM and SD-C contributed to the research, to the analysis of the results and to the writing of the manuscript.

## Conflict of Interest Statement

The authors declare that the research was conducted in the absence of any commercial or financial relationships that could be construed as a potential conflict of interest.

## Footnotes

^1^http://www.laskerfoundation.org/awards/show/deep-brain-stimulation-for-parkinsons-disease/. Accessed March 20, 2019.

^2^DBS market is evaluated around $700 million per year (and is projected to reach almost $1 billion for 2020): https://www.strategyr.com/market-report-deep-brain-stimulation-devices-forecasts-global-industry-analysts-inc.asp. Accessed March 20, 2019.

^3^Databases used: PubMed, Wiley on line, SAGE, Elsevier, BibCNRS.

^4^Main sources for animal rights associations: http://antidote-europe.org/; http://www.vero.org.uk/default.asp; http://www.fondation-droit-animal.org/; https://www.nonhumanrights.org/; http://www.animalexperiments.info/; http://www.international-campaigns.org/; https://one-voice.fr/. Accessed on March 20, 2019. Databases used: JStor, Cairn, BibCNRS, Doctrinal plus, Dalloz, Lexis-Nexis.

^5^All participants were fully informed of the research objectives and gave their informed consent.

^6^Some researchers had written alternative DBS histories that are exploring the role of industry or standardized clinical assessment tools in its development. Others focused on previous experiments in brain stimulation before Benabid’s discovery, or on the forms of practice that structure and promote the development and circulation of this neuromodulation technology: for example see Talan (2009); Hariz et al. (2010); Gardner (2013) and Moutaud (2016).

^7^http://www.laskerfoundation.org/awards/show/deep-brain-stimulation-for-parkinsons-disease/. Accessed March 20, 2019.

^8^Interestingly, Bailey and DeLong developed their arguments in subsequent publications (Bailey and Taylor, 2016; Wichmann et al., 2018).

^9^Please note that this article only focuses on the debates surrounding animal experimentation and animal models in DBS biomedical developments (i.e., experiments on animal models to explore and to understand normal and pathological human biology and to develop therapeutics). We will not discuss animal experimentation and animal models in fundamental biology or ethology for example.

^10^Our translation from French.

^11^Law No. 88-1138 of December 20th 1988, known as the “Huriet-Sérusclat law,” is the first French law devoted to the protection of persons involved in biomedical research.

^12^She also published in 2016 a commentary on the same topic with A. Ménache in *Frontiers in Human Neuroscience* (Ménache and Beuter, 2016).

^13^European Citizens’ Initiatives are instruments of participatory democracy, enabling citizens to propose legal changes in areas in which the European Commission is competent.

^14^www.stopvivisection.eu/sites/default/files/dossier_-11_may_2015.pdf. Accessed December 20, 2018.

^15^http://antidote-europe.org/en/jarrod-bailey-on-human-based-research/; http://antidote-europe.org/en/jarrod-bailey-on-gm-animals/. Accessed December 20, 2018.

^16^Bailey is also a member of *Cruelty Free International*, an association whose purpose is the protection of animals.

^17^Our translation from French.

^18^https://clinicaltrials.gov/ct2/show/NCT01984710. Accessed December 20, 2018.
